# A user application-based access point selection algorithm for dense WLANs

**DOI:** 10.1371/journal.pone.0210738

**Published:** 2019-01-16

**Authors:** Mun-Suk Kim, Yena Kim, SeungSeob Lee, SuKyoung Lee, Nada Golmie

**Affiliations:** 1 National Institute of Standards and Technology (NIST), Gaithersburg, MD, United States of America; 2 Department of Computer Science, Yonsei University, Seoul, Korea; RMIT University, AUSTRALIA

## Abstract

The current commercial access point (AP) selection schemes are mostly based on received signal strength, but perform poorly in many situations. To address this problem, a number of alternative schemes collect and analyze the actual load of every candidate AP. However, these schemes may incur significant latency and signaling overhead in dense wireless local area networks (WLANs). To mitigate such overhead, we propose a user application-based AP selection scheme that considers historical information about AP performance. Without inducing any signaling activity, our scheme monitors the amount of network traffic used by applications and estimates the achievable throughput of APs. Our scheme employs the characteristics of application traffic with the intent of accurately predicting AP performance. Using a measurement study in dense WLAN environments, we show that our scheme achieves higher throughput and lower association latency than those of existing schemes in places highly accessible to the user.

## 1 Introduction

Wireless local area network (WLAN) technology is an important player in providing reliable, portable and high-speed internet connectivity to end users [1]. To provide enough capacity for the large amount of traffic generated by the users, the density of access points (APs) gets much higher than that needed for coverage only [2]. However, high interference levels among APs can cause poor and unpredictable performances [3]. Unfortunately, since most of these APs are under decentralized control, it is complicated to share their resource according to the network condition [4]; therefore, the users commonly find tens of APs in each scan, which vary significantly with regard to the quality of internet connection [5].

The AP selection policy currently used by most existing IEEE 802.11-based products is based on the signal strength. However, this signal strength-based policy results in poor user experiences in many situations [5]. To solve this problem, a number of works have proposed additional metrics such as the bandwidth utilizations of APs, each individual device’s channel utilization, or the APs’ achievable throughput [5–13]; nevertheless, they have a few drawbacks. 1) Most of these schemes require either a designated server or special features at the APs to collect and analyze the bandwidth utilization and to distribute such statistics to mobile devices. The mobile users may additionally submit reports on the APs that they use; the privacy of user-submitted reports should be preserved. 2) Additional latency and signaling overhead are introduced to obtain the load information from every candidate AP in each scan. 3) A number of existing schemes overlook the APs’ backhaul capacities.

In this paper, to overcome the aforementioned problems, we propose a user application-based AP selection (UAAS) scheme based on the historical information about AP performance. Typically, a mobile user connects to a WLAN network mostly in a few places he/she frequently visits [14]. In these frequently visited places, our scheme collects the performance of each AP through the routine use of the WLAN service. When the user revisits the places, our scheme employs the collected performance to choose an appropriate AP. If the chosen AP does not meet the minimum bandwidth requirement while it is being used, a new association is preferred.

The major contributions of this paper are summarized as follows. 1) The proposed scheme does not incur any additional latency and signaling overhead. It monitors the network traffic trace of applications, which is simply managed by the mobile device, to collect the performance of the serving AP. This application traffic is the end-to-end throughput from the user to the service provider server; thus, our scheme can evaluate the backhaul capacities of the APs. 2) To accurately predict AP performance, our scheme classifies application traffic into three different types and considers the characteristics of each traffic type. 3) Our scheme does not require any modifications to the AP, nor does it require a designated server.

In addition to the above contributions, we have conducted measurement studies. We identified locations where APs were deployed densely and then investigated the performance of these APs. We also analyzed the features of each type of application traffic and the relationship between user mobility and WLAN connectivity. Our measurement studies have been utilized to evaluate the proposed scheme and the legacy association schemes presented in [5, 15].

The remainder of this paper is organized as follows. Section 2 briefly explains the related work. Section 3 presents the results of our measurement study. Our UAAS scheme is detailed in Section 4. Section 5 discusses the performance evaluation results. Finally, Section 6 concludes this paper.

## 2 Related work

The proposed scheme is related to two research areas: AP selection and application traffic classification.

### 2.1 AP selection

The signal-strength-first (SSF) scheme selects the AP with the best signal-to-noise ratio (SNR) [15]; however, it overlooks the actual load distributions among APs. This problem has been addressed extensively in the literature with two different approaches: distributed and centralized [5–13].

The distributed solution measures AP performance in two ways. First, the mobile device obtains the measurements from the network. For instance, in the IEEE 802.11k/v amendments [6], it requests link quality information from other mobile devices or candidate APs. The scheme in [5], i.e., Virgil, associates with every candidate AP and tests its expected bandwidth by communicating with a designated server. The scheme in [7] probes every candidate AP and computes its achievable bandwidth according to the time spent exchanging the probing frames. However, these active measurement schemes demonstrate additional latency and signaling overhead to examine every candidate AP, and it becomes more critical in dense WLANs where tens of APs are deployed in close proximity to each other. Second, the mobile device observes the transmissions of other mobile devices or candidate APs to estimate the load of each basic service set (BSS) [8–10]. However, all of these passive observation schemes ignore the backhaul capacities of candidate APs.

The centralized solution relies on the global view obtained from the network controller. The scheme in [11] analyzes the load conditions of APs and evenly distributes mobile users among the APs. This scheme requires considerable modifications at the APs to control the user associations and balance the AP loads. The scheme in [12], i.e., Wifi-Reports, collects the historical information about AP performance in a manner similar to our scheme. However, unlike our scheme, Wifi-Reports needs a designated server and mobile devices consume additional battery power to submit reports on the APs that they use. More recently, the scheme in [13] takes advantage of the flexibility and centralized nature of software-defined networking (SDN). The SDN controller considers the quality of service requirement of a user joining the network, the current network capacity, and the quality of the connectivity provided to the remaining users. However, this scheme consume substantial signaling overhead to keep track of all the flows connected to the network.

### 2.2 Application traffic classification

Network traffic classification schemes can be grouped into three categories: 1) port-based, 2) payload inspection, and 3) statistical traffic properties [16–20]. Port-based classifiers extract the port number of the packet header, which is associated with a particular application. The payload inspection schemes, known as deep packet inspection, look for application-specific data within the packet payload. Unlike these two approaches, the schemes based on statistical traffic properties recognize externally observable characteristics of the traffic, e.g., inter-packet arrival times. Our proposed scheme considers the characteristics of application traffic to estimate AP performance; thus, we focus on the statistical machine learning schemes in this paper.

The scheme in [16] utilizes the Bayesian analysis technique. This naive Bayes method is tractable because of its computational simplicity; however, it achieves an accuracy of only 65% for per-flow classification. This scheme can be incorporated with the kernel density estimation theory, called naive Bayes kernel (NBK), to improve its classification accuracy to over 95%. The decision tree-based scheme in [17] and the Bayesian neural network (BNN) schemes in [18] and [19] consist of two processes: 1) a training process for tree or neural network construction, and 2) a testing process for classification decision. Such approaches have low time complexity in the testing process, satisfying good classification accuracy. In particular, the BNN schemes can classify flows with up to 99% accuracy [20]; however, they require high time complexity in the training process. This is because, using the training data set, the BNN schemes have to search for all the link weights between the nodes comprising a neural network. In Section 5, our scheme adopts the NBK-based classifier because it is a simple computational method that works better than the more complex ones.

## 3 Preliminary study

We conducted measurement studies to explore the characteristics of dense WLANs and user application traffic and to analyze the effect of user mobility on WLAN connectivity.

### 3.1 Measurement study in dense WLANs

**Fig 1:**
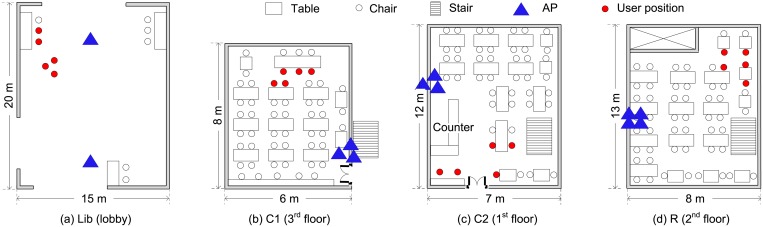
Floor plans of (a) Lib, (b) C1, (c) C2, and (d) R.

For our field study setting, we developed an Android mobile application, called Field Study APplication (FSap). We set up a designated server at the 3rd Engineering Building in Yonsei University in Seoul, Korea, and uploaded a 100 Mbyte text file. Specifically, FSap scans for all available APs and then measures each AP’s achievable throughput by downloading the text file in the same manner as in [5]. After completing the association with the AP, FSap establishes a Transmission Control Protocol (TCP) connection with the designated server and receives the text file at full speed for 20 s. The field study was performed in four locations that people often visit near Yonsei University: the library (Lib), cafe 1 (C1), cafe 2 (C2), and restaurant (R). These locations offer the WLAN services of three service providers (SPs) in addition to their private ones. In Korea, each SP shares most of its own APs so that the subscribers of other SPs can use them free of charge. Fig 1 shows the floor plan of each location; we have marked the positions of the user and APs. Although the exact same position was not used every day, the user used one of the five seating positions located near each other in each location. All the APs found in a scan could not be marked in Fig 1; some APs were hidden and some APs were located on different floors or even in different buildings. The commercial APs of the three SPs were located together in C1, C2, and R. Wireless routers, which are normally used as commercial APs, simultaneously use both 2.4 GHz band for 802.11 b/g/n and 5 GHz band for 802.11 a/n, with different BSS identifiers (BSSIDs); their channel width can be configured to either 20 or 40 MHz [21].

We used Nexus 7 tablets supporting dual-band 802.11 a/b/g/n connection; our mobile devices can be associated with all commercial APs, unencrypted private APs and some encrypted private APs for which our mobile devices hold their encryption keys. The time of day ranged from 9 am to 10 pm was quantized into two or four time intervals, each lasting six or three hours, respectively. We collected two measurements per AP, every hour for four weeks.

**Fig 2:**
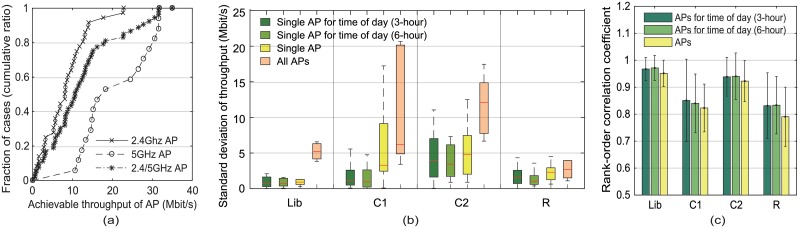
Cumulative distribution functions, standard deviation, and rank-order correlation coefficient. (a) cumulative distribution functions for the achievable throughputs of usable APs and the (b) standard deviation and (c) rank-order correlation coefficient of these achievable throughputs at each location.

Table 1 gives the statistics for the APs found at a specified seating position within each location. Every AP was identified by its unique BSSID. We define a usable AP as one that grants an IP address to the mobile device, and allows the file download from the designated server. Fig 2(a) shows the cumulative distribution functions for the average achievable throughput of usable APs deployed in all locations. Fig 2(b) illustrates the distributions for the standard deviation of the achievable throughput of all usable APs deployed in each location, of each single usable AP, and of each single usable AP for the time of the day. When we considered the time of the day the throughput was measured, the standard deviation was calculated for each predetermined time period, i.e., three or six hours; otherwise, it was computed for each day. We found that there was a significant range in the achievable throughput of APs in each location; however, the variance for a single AP was much smaller than this wide performance range. To test whether the rank order among the APs is well preserved, we employ the Spearman rank-order correlation coefficient (SRCC) introduced in [22]. Fig 2(c) illustrates the rank-order similarity among the achievable throughput sets for APs in each location. The SRCC ranges from + 1 to −1, and it moves closer to + 1 as the similarity increases. It shows high SRCCs of more than 0.8 across different locations. Better SRCC results were found when the measurement time of day was considered. However, there was little difference between quantizing the time of the day every three hours and quantizing the time of the day every six hours.

**Table 1:** AP statistics in our field study.

Location	Radio frequency (GHz)	APs found	Private APs (usable #)		Commercial APs (usable #)			Usable APs
			Unencrypted	Encrypted	SP1	SP2	SP3	
Library	2.4	6	0 (0)	6 (4)	0 (0)	0 (0)	0 (0)	4 (66.7%)
	5	6	0 (0)	6 (5)	0 (0)	0 (0)	0 (0)	5 (83.3%)
Cafe 1	2.4	11	3 (1)	0 (0)	8 (8)	0 (0)	0 (0)	9 (81.8%)
	5	7	3 (2)	0 (0)	4 (3)	0 (0)	0 (0)	5 (71.4%)
Cafe 2	2.4	25	5 (4)	2 (1)	12 (9)	3 (2)	3 (0)	16 (64%)
	5	13	3 (1)	0 (0)	7 (6)	2 (1)	1 (0)	8 (61.5%)
Restaurant	2.4	22	4 (0)	1 (1)	2 (2)	9 (6)	6 (1)	10 (45.5%)
	5	11	0 (0)	2 (0)	6 (2)	0 (0)	3 (0)	2 (18.2%)

Thus, in dense WLANs, our results suggest the following: 1) The APs vary considerably with regard to their achievable throughput. 2) Their historical achievable throughput is useful enough to compare the candidate APs especially when we consider the time of day that it was measured.

### 3.2 Analysis of application traffic

We analyzed the traffic of widely-used applications. To this end, we surveyed 87 users in Seoul, Korea, including 31 office workers and 56 graduate and undergraduate students. We investigated what applications the participants used at least once a week, how often they used each application, and how long they used each application at any given time.

In Android, an application comprises one or more packages, and the amount of network traffic used by each package is recorded with its unique ID; therefore, in our experiments, the applications were considered on a per package basis. We manually grouped together packages with similar names and descriptions that were presented in the Android application market, among those of the surveyed applications. For example, an application called Daum, which is an Android version of the web portal, offers online map and webtoon services; its packages, net.daum.android.map and net.daum.android.webtoon, were grouped into online maps and webtoons, respectively, in Table 2. Those package groups were classified into three different types: streaming, browsing, and download [23].

**Table 2:** Applications for traffic analysis study.

Type	Application
Streaming	Streaming music and video, real-time television, Voice Over IP, internet radio services
Browsing	Web browsers, online maps, news, and magazines, webtoons, social networking services
Download	Application stores, system application for updates, file downloads, cloud services, P2P applications

To obtain the measurements, we developed an Android application that monitors the throughput of each application package currently running in the mobile device. We chose one or two packages per package group presented in Table 2, and for each chosen package, our monitoring application checked its traffic every 0.25 s; if there was a change in the amount of traffic received by the package, our monitoring application calculated the throughput for 0.25 s. We installed an AP that supports IEEE 802.11g with a channel width of 20 MHz. To configure the downlink bandwidth, we accessed the AP’s utility via a web browser on a computer connected to the AP and set its transmission rate to 1, 2, 5.5, 11, or 54 Mbit/s. For each downlink bandwidth, we measured the throughput of application packages included in each type to see if the actual throughput of the AP can be estimated from the measured application throughput. We defined the actual throughput of an AP as the maximum end-to-end throughput that a user can achieve through the AP; the AP’s actual throughput depends on multiple factors such as the location and communication capability of a destination service server. In our experiments, we measured the actual throughput of APs through a commercial internet speed test (IST) website while the APs were being used by the mobile device [24]. We collected 500 traffic samples per application package at each downlink bandwidth.

**Fig 3:**
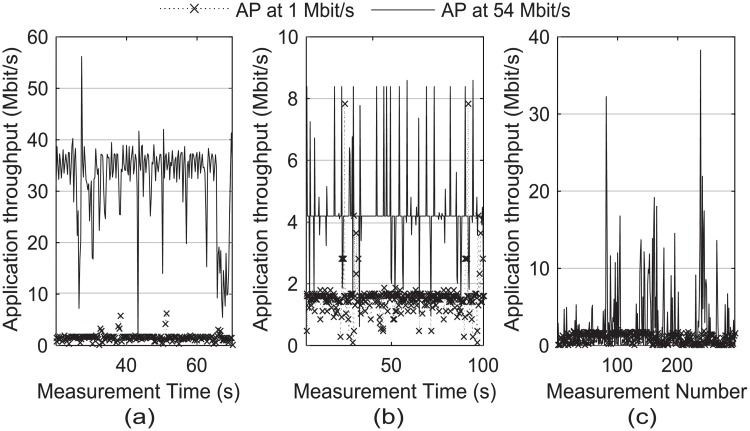
Throughput traces of the application packages. (a) the file download, (b) YouTube, and (c) the web browser, when the downlink bandwidth was configured to 1 and 54 Mbit/s.

Fig 3 shows a sample throughput trace for each application type. We used the file download from a popular website, YouTube, and the default web browser as example applications of download, streaming, and browsing types, respectively. In Fig 3(c), we used the measurement number as the x-axis because the web browsing traffic is bursty in nature. We found that all of these traffic samples were different enough to distinguish between the downlink bandwidths of 1 and 54 Mbit/s.

**Fig 4:**
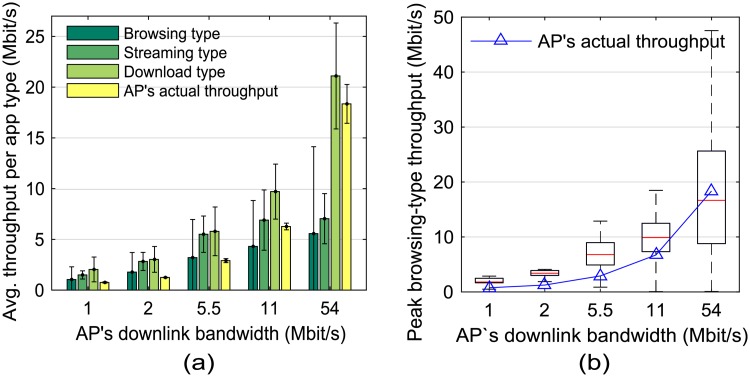
Average and peak throughput. (a) averages of the throughput and their standard deviations when applications of browsing, streaming, and download types were used. (b) Peak throughput of browsing-type applications.

Fig 4(a) shows that the download-type throughput increased as much as the available bandwidth increases, whereas the streaming-type throughput remained almost constant if the available bandwidth was greater than its bandwidth requirement. We define the normalized standard deviation as the ratio of the standard deviation of the collected traffic to its average throughput. Table 3 shows that, on average, the normalized standard deviation was 71% and 67% higher in the browsing type than in the streaming and download types, respectively. This is because the browsing-type throughput fluctuates widely according to web contents, e.g., only text or graphics. Fig 4(b) shows that the mean peak throughput of the browsing type was close to the AP’s actual throughput. We chose the highest one from among 20 traffic measurements as the peak throughput.

**Table 3:** Normalized standard deviation of each application type.

AP’s bandwidth (Mbit/s)	1	2	5.5	11	54
Browsing	1.19	1.08	1.17	1.05	1.54
Streaming	0.27	0.31	0.32	0.43	0.35
Download	0.6	0.42	0.41	0.28	0.25

Therefore, the characteristics of each traffic type can be summarized as follows: 1) The average throughput was higher in the download type than in the other types, whereas the normalized standard deviation was highest in the browsing type. 2) The download type was most accurate for ranking AP performance. 3) In the browsing type, the mean peak throughput was closer to the actual AP capacity than the average throughput. These characteristics were evident especially when the mobile device was associated with the high-speed AP.

### 3.3 Analysis of user mobility and connectivity

We collected the cellular connection information from 70 users consisting of 39 students and 31 office workers. These participants used our monitoring application, WiNet [25], for two months on their primary mobile phones equipped with Long-Term Evolution (LTE) and WLAN interfaces. WiNet continuously monitored the LTE cell identifier (CID), tracking area code (TAC), and AP association information at every 10 s and stored these pieces of information only if there was a change between the previous and the new ones.

**Fig 5:**
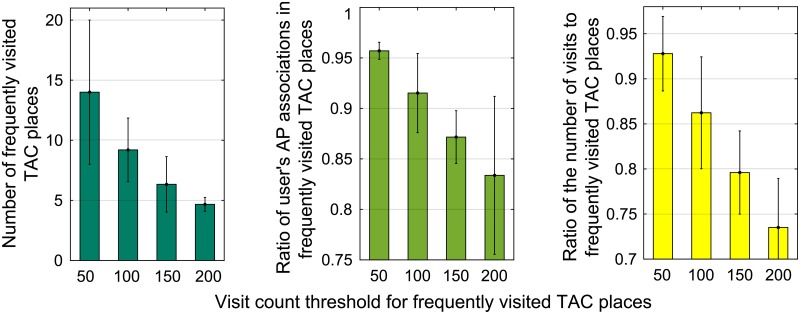
User mobility and WLAN connectivity. (a) the number of frequently visited TAC places and (b) the ratio of AP associations and (c) visits that a user did in the frequently visited TAC places.

We analyzed 244 041 monitoring records in total. On average, the cellular association information of each user included 227.3 LTE CIDs and 43.7 TACs, and the user was associated with 7.35 LTE cells and 8.1 APs in the place identified by a TAC. Fig 5 shows the patterns of user mobility and WLAN connectivity in TAC places frequently visited by the users. We found that much fewer TAC places were classified as frequently visited ones when the visit count threshold was 100 than when it was 50; nevertheless, the users still did most of their AP associations and visits in the frequently visited TAC places when the visit count threshold was 100.

These results are explained as follows: 1) The visited places could be identified by the cellular connection information, i.e., TAC. 2) There existed a few places that the user frequently visited, and most of his/her AP associations were performed in these frequently visited places.

## 4 User application-based AP selection scheme

On the basis of the observations in the previous section, we propose a UAAS scheme that considers the historical information about AP performance. The goals of our scheme are as follows. First, it accurately examines the achievable throughput of each accessible AP which will be used as the historical information. Second, our scheme mitigates the latency and signaling overheads for obtaining the historical information in dense WLANs.

In this section, we describe the basic concept of the proposed scheme and then present the technical details. Table 4 summarizes the notations used in the rest of the paper.

**Table 4:** List of parameters and descriptions.

Notation	Description
*ψ*	The similarity threshold between WLAN vectors scanned.
Ω	The set of candidate APs scanned by the mobile device.
A	The set of application types.
*t*_*s*_, *t*_*b*_, *t*_*d*_	The streaming, browsing, and download types, respectively.
*n*_*l*_	The number of times that the user visits a place *l*.
*n*_*φ*_	The total number of application throughput measurements that have been stored as the historical information of AP *φ*.
$n_{t}^{\left(\varphi \right)}$	The number of application throughput measurements for type *t*, which have been stored as the historical information of AP *φ*.
*δ*_*v*_	The predefined visit count threshold.
*δ*_max_	The maximum number of all types of application throughput measurements to be stored in the DB.
$\delta_{\min}^{\left(t\right)}$	The minimum number of application throughput measurements for type *t* to be stored in the DB.
*δ*_*b*_	The lower bound of AP’s achievable throughput that satisfies the maximum bandwidth requirement of user applications.
*B*_*φ*_	The predicted achievable throughput of AP *φ*
*D*_max_	The upper bound of the time in which our scheme postpones the AP association switching.

### 4.1 System overview

**Fig 6:**
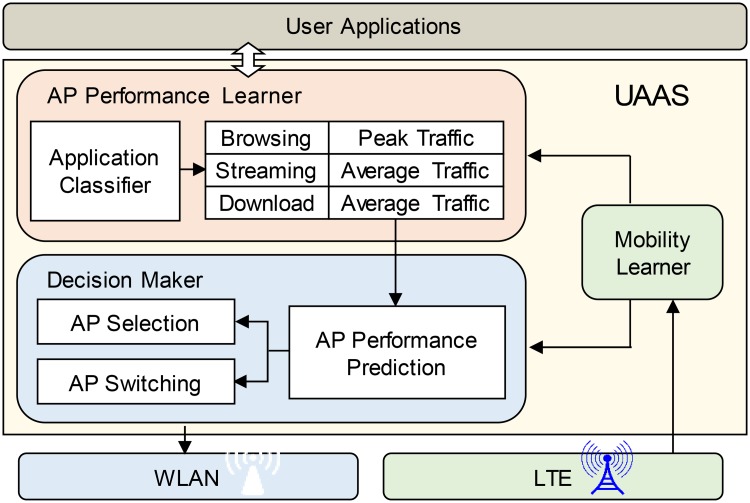
Architecture of our UAAS system.

We assume that mobile devices are equipped with LTE and WLAN interfaces. The proposed scheme comprises the mobility learner, AP performance learner, and decision maker, as shown in Fig 6. The mobility learner manages the places visited by a user. We define a *visited place* as the area identified by the LTE connection, i.e., TAC. In the frequently visited places, the AP performance learner collects the achievable throughput of the AP that is being used, without inducing any signaling exchanges with the network. To this end, our scheme monitors the traffic of active applications running on the mobile device. Our scheme classifies application traffic into three types, i.e., browsing, streaming, and download.

When choosing an AP in a visited place, our scheme utilizes the historical performance of the candidate APs in a scan set. The decision maker predicts their achievable throughput by considering the characteristics of each type of application traffic, the time of day that the historical performance was measured, and the SNRs from the APs; it then chooses the AP for which the highest achievable throughput has been predicted. If the throughput of the chosen AP is measured to be lower than the minimum bandwidth requirement while it is being used, the decision maker changes its association to an AP that offers more bandwidth.

### 4.2 AP performance learning

When the user stays in a visited place for a certain time, our scheme stores the signatures of the visited place, i.e., LTE CID, TAC, and visit time, in a local database (DB). In order to preserve the privacy of the user’s place, it should be ensured that the signatures of the visited places in the DB can only be managed by the system and are not accessible to the applications [26]. Our scheme incrementally constructs the visited places in daily life and recognizes the revisited places from their signatures. Let *n*_*l*_ and *δ*_*v*_ denote the number of times that the user visits a place *l* and the predefined visit count threshold, respectively. If *n*_*l*_ > *δ*_*v*_, we define the place *l* as a place frequently visited by the user. Based on our analysis of user mobility and connectivity in Section 3.3, the value of *δ*_*v*_ was set to 100 in the rest of the paper.

When a user device is unable to use the LTE interface, our scheme recognizes a room-level visited place. Each place can be identified by the signaling fingerprints of the surrounding APs [27]. Let ${\vec{F}}_{l_{1}}$ and ${\vec{F}}_{l_{2}}$ denote the list of SNR values of beacons sent by the surrounding APs at places *l*_1_ and *l*_2_, respectively. Then, the WLAN similarity function is defined using the Tanimoto Coefficient, as follows:

$$\begin{array}{c} \begin{array}{c} S\left({\vec{F}}_{l_{1}},{\vec{F}}_{l_{2}}\right)=\frac{{\vec{F}}_{l_{1}}\cdot {\vec{F}}_{l_{2}}}{∥{\vec{F}}_{l_{1}}{∥}^{2}+{∥{\vec{F}}_{l_{2}}∥}^{2}-{\vec{F}}_{l_{1}}\cdot {\vec{F}}_{l_{2}}} \end{array} \end{array}$$ (1)

Let *ψ* denote the similarity threshold. If $S({\vec{F}}_{l_{1}},{\vec{F}}_{l_{2}})>\psi$, *l*_1_ and *l*_2_ are considered as the same place.

At the frequently visited places, our scheme examines AP performance by monitoring the traffic of active applications currently running in the mobile device. In the Android framework, the amount of internet traffic received by each application is automatically recorded in a system file named “/proc/uid_stat”. The application traffic is grouped into three types: streaming, browsing, and download [23]. We use two families of traffic classifier: NBK-based and BNN-based [20]. The BNN-based method is more accurate than the NBK-based one, but has a higher time complexity in the training process, as described in Section 2. Thus, the BNN-based classifier is suitable for high-end mobile devices.

As noted in Section 3.2, each type of application traffic exhibits different characteristics in terms of its average and normalized standard deviation, which are denoted by *D*_*a*_ and *D*_*v*_, respectively. Therefore, we refer to these two attributes as the discriminators of our classifier. Consider a data sample **x** = {*x*_1_,…, *x*_*n*_} where each data element *x*_*i*_ is described by two discriminators, {*D*_*a*_, *D*_*v*_}. Then, it is given by $x_{i}={\left(D_{a}^{\left(i\right)},D_{v}^{\left(i\right)}\right)}^{T}$. Let $A=\{t_{s},t_{b},t_{d}\}$ be the set of application types, where *t*_*s*_, *t*_*b*_, and *t*_*d*_ represent the streaming, browsing, and download types, respectively. For each collected data element *x*_*i*_ in set **x**, we can define a mapping function $T\left(x_{i}\right)=t\left(t\in A\right)$. This means that the data element *x*_*i*_ belongs to the application type *t*.

The technical details of our NBK-based traffic classifier can be explained as follows. Let *n*_*t*_ denote the number of collected data elements belonging to the type t(t∈A), given a training data set **x** = {*x*_1_, …, *x*_*n*_}. Then, the probability of obtaining the type *t* independent of the observed data, is

$$\begin{array}{c} \begin{array}{c} p\left(t\right)=\frac{n_{t}}{n} \end{array} \end{array}$$ (2)

where $n=\sum_{t\in A}n_{t}$.

Let $y={\left(D_{a}^{\left(y\right)},D_{v}^{\left(y\right)}\right)}^{T}$ be a newly observed data element, and let $\hat{f}\left(y|t\right)$ be the distribution function for the probability that *y* belongs to the application type *t*, given a training data set, **x**. Then, $\hat{f}\left(y|t\right)$ is estimated using the Gaussian density as the kernel for the analysis. With *H* denoting the 2 × 2 bandwidth matrix of the kernel, we thus have

$$\begin{array}{c} \begin{array}{c} \hat{f}\left(y|t\right)=\frac{1}{n_{t}}\cdot \sum_{x_{i}:T\left(x_{i}\right)=t}K\left(y-x_{i}\right) \end{array} \end{array}$$ (3)

where $x_{i}={\left(D_{a}^{\left(i\right)},D_{v}^{\left(i\right)}\right)}^{T}$ and *K*(⋅) is the kernel of our classifier which is given by

$$\begin{array}{c} \begin{array}{c} K\left(\text{Y}\right)=\frac{1}{\sqrt{2\pi \cdot |H|}}\cdot \exp(\frac{-{\text{Y}}^{T}H^{-1}\text{Y}}{2}) \end{array} \end{array}$$ (4)

where $\text{Y}=y-x_{i}$ and ∫K(Y)dY=1.

**Fig 7:**
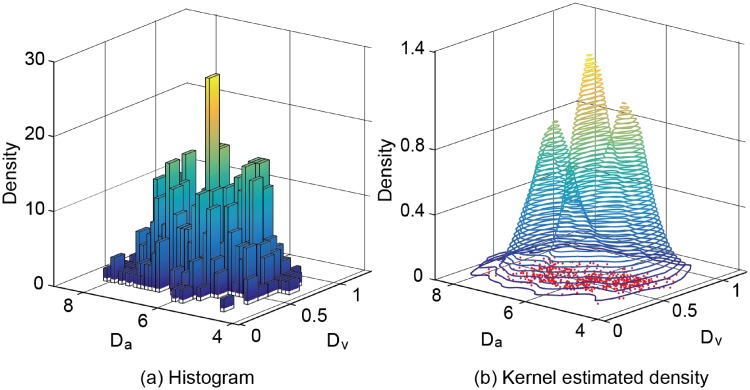
Examples of (a) the histogram and (b) the kernel estimated density. Given 600 training instances for the average and the normalized standard deviation of the streaming-type application traffic, i.e., *D*_*a*_ and *D*_*v*_, respectively.

In Eq (4), the kernel bandwidth matrix, *H*, has a significant effect on the classification accuracy. It is symmetric and positive definite, and is obtained on the basis of the mean integrated squared error as follows [16]:

$$\begin{array}{c} \begin{array}{c} MISE\left(\hat{f}\right)=E[\int {\left(\hat{f}\left(x|t\right)-f\left(x|t\right)\right)}^{2}dx] \end{array} \end{array}$$ (5)

where *f*(⋅|*t*) is a histogram for the given training data set. Fig 7 illustrates an example of estimating the density, $\hat{f}\left(\cdot |t\right)$. We selected three streaming-type applications from Table 2 and measured their traffic at AP’s downlink bandwidth of 54 Mbit/s. We then collected 600 training instances for the average and the normalized standard deviation of their traffic. The figure shows that the kernel estimated density with the bandwidth matrix $H=\left(\begin{array}{cc} 0.011 & 0.004\\ 0.004 & 0.011 \end{array}\right)$ is close to the histogram of the given training instances.

Let *p*(*t*|*y*) denote the probability that a newly observed element *y* belongs to an application type *t*. Then, using Eqs (2) and (3) and referring to the Bayes’ theorem, we obtain

$$\begin{array}{c} \begin{array}{c} p\left(t|y\right)=\frac{p\left(t\right)\hat{f}\left(y|t\right)}{\sum_{t\in T}p\left(t\right)\hat{f}\left(y|t\right)} \end{array} \end{array}$$ (6)

Thus, using Eq (6), our NBK classifier can determine the type of a newly observed data element *y* as follows:

$$\begin{array}{c} \begin{array}{c} t^{\left(y\right)}=\arg\max_{t\in T}p(t|y)\;\\ \text{Subject}\;\text{to}\;\;\;p\left(t|y\right)>\delta_{p} \end{array} \end{array}$$ (7)

where *δ*_*p*_ is the predetermined threshold for traffic classification. If *p*(*t*|*y*) ≤ *δ*_*p*_, our scheme excludes, from the process of AP performance learning, the application that has generated the data element *y*.

The BNN-based classifier uses multilayer perceptron networks [18]. The first layer contains the inputs, which, in our problem, are the two discriminators ${\left(D_{a}^{\left(i\right)},D_{v}^{\left(i\right)}\right)}^{T}=x_{i}$. The final layer contains the outputs, and, in our problem, each output is the probability that the input data *x*_*i*_ belongs to each application type in set A. Intervening layers are referred as to *hidden*. Each hidden layer can comprise any number of nodes, and the nodes are connected by weighted links to all nodes in adjacent layers. Given the weights for the neural network, the nodes calculate their *activations*. Let *w*_*ij*_ and **w** = {*w*_*ij*_} be the *weight* from node *i* to node *j* and the set of weights for the neural network, respectively. Then, the activation of node *j* is *u*_*j*_ = ∑_*i*_ *w*_*ij*_ *g*(*u*_*i*_), where *g*(⋅) is the hyperbolic tangent activation function, i.e., *g*(*u*_*i*_) = tanh(*u*_*i*_). In our problem, we apply the *softmax filter* to the output layer so that its activations behave like a probability distribution. Thus, the activation of the *j*^th^ node in the output layer is *u*_*j*_ = exp(*v*_*j*_)/∑_*k*_exp(*v*_*k*_), where *v*_*j*_ = ∑_*i*_ *w*_*ij*_ *u*_*i*_. Let *p*(*t*|**w**, *y*) be the probability that a newly observed element *y* belongs to type *t* given a set of parameters **w**. Then, using the activations in the output layer, we set *p*(*t*|**w**, *y*) = *u*_*j*_ for *j* = *t*. Finally, the type of a newly observed element *y* can be determined by substituting *p*(*t*|**w**, *y*) for *p*(*t*|*y*) in Eq (7).

We note that the characteristics of each type of application traffic are obvious when the high-speed AP is used as described in Section 3.2. Therefore, our classifier operates when the currently connected AP has a high achievable throughput. To see if the current AP is enough to operate the classifier, we evaluate the throughput of predetermined applications, e.g., “android.process.media,” which is a system process for downloads in the Android framework.

Then, the AP performance learner collects the achievable throughput of the current AP using the following procedure:

1. If the number of times that the user visited place *l*, *n*_*l*_, is lower than the predetermined threshold, i.e., *n*_*l*_ < *δ*_*v*_, our scheme increases *n*_*l*_ by 1 and terminates the learning process; otherwise, it measures the performance of the AP currently being used by the user.
2. To collect AP performance, our scheme monitors the amount of traffic received by each active application and then computes its throughput periodically. If the application belongs to the browsing type, our scheme obtains the peak throughput from among multiple consecutive throughput measurements.
3. These application throughput measurements are stored in the local DB.

The historical information about AP performance may not be valid over time owing to backhaul and AP upgrades. To ensure the freshness, our scheme defines the time-to-live (TTL) value and removes each historical information from the DB when its TTL expires.

Although we use the downlink traffic received by each application to examine the downlink performance of an AP, our proposed learning process can be easily extended to the monitoring of uplink traffic in order to investigate the AP’s uplink performance. In Android, the amount of uplink traffic sent by each application is recorded in the same system file as that used for the downlink traffic.

### 4.3 User application-based AP selection

The expected performance of each AP varies according to the time of the day and the wireless channel conditions; that is, the throughput of the AP is likely to be low during the time of the day when the users are crowded and the loss rate of a wireless channel is roughly inversely proportional to the SNR [28]. To preserve accuracy, our UAAS scheme measures the application throughput as described in Section 4.2, and then stores, in the DB, each application throughput annotated with the median SNR of the beacons sent by the serving AP and with the time of the day when it was measured [12]. When the user re-encounters the AP, our scheme only considers the historical application throughput values taken in the same SNR and time-of-day range as that of the currently observed SNR and time-of-day.

**Fig 8:**
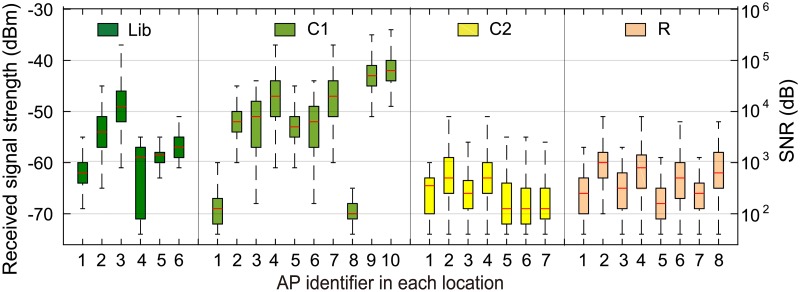
Received signal strength/SNR of usable APs in each location.

Specifically, the SNR values are partitioned into three ranges according to the wireless loss: ranges where the user experiences nearly 100% loss, intermediate loss, and nearly 0% loss [12]. In Section 5, the intermediate loss range is scaled to an SNR interval of [22, 32] dB [12]. Fig 8 shows the received signal strength/SNR of the usable APs that were always found in each location during the field study described in Section 3.1. It was assumed that the environment noise was fixed at -90 dBm [29]. The time of the day when the application throughput is measured is quantized into *n*_*I*_ time intervals, each lasting 24/*n*_*I*_ hours. The achievable throughput of the APs in the temporal aspect is related to the locations where those APs are deployed; in each location, the users are crowded at different times of the day. Therefore, our scheme reflects the local time in the time zone associated with each location.

Algorithm 1 presents the pseudocode of the decision maker, where the required achievable throughput for AP selection, *δ*_*b*_, is needed to avoid the unnecessary learning iterations. For example, let us suppose that the mobile device has scanned four APs whose achievable throughput is AP1 = 5 Mbit/s, AP2 = 22 Mbit/s, AP3 = 25 Mbit/s, and AP4 = 28 Mbit/s, respectively. If the maximum bandwidth requirement of its applications is *δ*_*b*_ = 20 Mbit/s, the mobile device will be satisfied with any of AP2, AP3, and AP4. Therefore, our scheme performs the learning process until it has investigated the achievable throughput of one of AP2, AP3, and AP4.

**Algorithm 1 User application-based AP selection**

**Output**: Selected candidate AP *φ*

**Notation**: Set of candidate APs Ω, required achievable throughput for AP selection *δ_b_*, Cell connection information *l*, visit count threshold *δ*_*v*_

1: Ω ← getSetOfCandidateAP();        # (1)

2: *l* ← getCellularConnectionInfo();       # (2)

3: *n*_*l*_ ← getVisitCount(*l*);           # (2)

4: **if** *n*_*l*_ < *δ*_*v*_ **then**               # (2)

5:  LegacyAPselection(Ω);          # (2)

6:  **return**

7: **end if**

8: **for each** candidate AP *φ* ∈ Ω **do**

9:  **if** NeedAPLearning(*φ*) **or**         # (3)

10:    PredictAPperformance(*φ*) > *δ*_*b*_ **then**  # (4)

11:   **return *φ***

12:  **end if**

13: **end for**

14: *φ* ← arg max_*φ*′∈Ω_{PredictAPperformance(*φ*′)}  # (5)

15: **return** *φ*

The detailed procedure of AP selection proceeds as follows:

1. Given a set of candidate APs, Ω, our scheme sorts them by their last access time. If some candidate APs have never been used before, they are sorted in descending order by their beacon SNRs.
2. We then recognize the current visited place *l* from the LTE connection. If the user has occasionally visited the place *l*, i.e., *n*_*l*_ < *δ*_*v*_, our scheme uses one of the legacy AP selection approaches, such as SSF and the probing of candidate APs presented in Refs. [5, 7].
3. In the frequently visited places, i.e., *n*_*l*_ ≥ *δ*_*v*_, we check whether the historical information of each candidate AP *φ* has been collected enough to predict its achievable throughput. Specifically, referring to the notations in Table 4, if *n*_*φ*_ < *δ*_max_ or $n_{t}^{\left(\varphi \right)}<\delta_{\min}^{\left(t\right)}$ for at least two types, our scheme chooses the AP *φ* to fully obtain its historical information.
4. Otherwise, we predict the achievable throughput of the AP *φ*. Note that the download type is the most reliable for ranking the qualities of the candidate APs. Thus,
  - if the download-type measurements are sufficient, i.e., $n_{t_{d}}^{\left(\varphi \right)}\geq \delta_{\min}^{\left(t_{d}\right)}$, the achievable throughput is the average throughput of this type of applications;
  - otherwise, all types of application throughput are averaged as follows:
    $$\begin{array}{c} \begin{array}{c} B_{\varphi }=\frac{1}{\left|A\right|}\sum_{t\in A}\tau_{t}^{\left(\varphi \right)} \end{array} \end{array}$$ (8)
    where $\tau_{t}^{\left(\varphi \right)}$ is the average of the *t*-type application throughput that has been measured as the historical information of the AP *φ*.
  If the achievable throughput is above the maximum bandwidth requirement, i.e., *B*_*φ*_ > *δ*_*b*_, our scheme associates with the AP *φ*; otherwise, it repeats steps 3 and 4 for the next candidate AP in set Ω.
5. If the achievable throughput of every candidate AP is lower than the threshold *δ*_*b*_, we choose the AP for which the highest achievable throughput has been predicted.

Additionally, we include the following APs in the blacklist: open APs that do not grant a dynamic host configuration protocol (DHCP) address, APs that selectively block traffic according to the medium access control (MAC) addresses, and APs that redirect traffic to their own servers. The blacklisted APs are excluded from the procedure of Algorithm 1.

### 4.4 User application-based AP switching

When the measured application throughput is lower than the minimum bandwidth requirement, e.g., the minimum bandwidth requirement for online video is 1 Mbit/s [7], our scheme decides to switch its association. However, if a set of mobile devices switches to the same AP simultaneously, these mobile devices would still experience poor throughput performance at the newly associated AP. To address this problem, we propose an AP switching policy based on the *probabilistic* and *delayed* switch. The detailed procedure is as follows:

1. Once the average application throughput has been measured below the predetermined minimum requirement, our scheme postpones the switching by *T*_*delay*_, which is randomly selected between [0, *D*_max_]. During *T*_*delay*_, it keeps measuring the throughput of the active applications. After *T*_*delay*_, our scheme switches if the average application throughput remains below the minimum bandwidth requirement.
2. If there exist some candidate APs whose performance have not been collected, our scheme switches to one of these unexamined APs with the same probability, 1/*k*, where *k* is the number of the unexamined APs.
3. Otherwise, if the performance of all candidate APs have been collected, our scheme probabilistically switches to one of these examined APs. The probability of switching to a candidate AP *φ* is proportional to its achievable throughput; thus, it is given by *B*_*φ*_/∑_*φ*′∈Ω_ *B*_*φ*′_.

To avoid unnecessarily bouncing back and forth, our proposed scheme restricts the maximum number of times that a mobile device can switch its association in a location. When leaving the location, the mobile device resets the number of counted switchings. A change in location can be detected using Eq 1. Let ${\vec{F}}_{\kappa }$ be the signaling fingerprints of the surrounding APs at time *κ*. Then, if $S({\vec{F}}_{\kappa -1},{\vec{F}}_{\kappa })\leq \psi$, our scheme recognizes the change in location between times *κ* − 1 and *κ*.

## 5 Performance evaluation

In this section, we evaluate the performance of the proposed AP association scheme. We utilized our ground-truth measurements to simulate the following two scenarios:

- Scenario 1: The user position was fixed in an area where five APs were used; their downlink bandwidth was configured to 1, 2, 5.5, 11, and 54 Mbit/s, respectively.
- Scenario 2: We deployed 10, 7, 6, and 8 APs in C1, C2, Lib, and R presented in Section 3. Five seating positions were defined in every location, as shown in Fig 1.

For each AP in these scenarios, we conducted a measurement study in the same way as that in Section 3. We selected eight applications per type. For each application, we measured the traffic every 0.25 s and computed the average and the normalized standard deviation of each set of 50 traffic samples. In addition to application traffic, we collected the following measurements at each user position over two weeks: SNR and actual throughput obtained by the IST website [24]. We managed 331 791 measurement sets in total and randomly used them as the historical information of the APs. Two types of periods were defined for each day: daytime between 9 am and 6 pm and nighttime between 6 pm and 10 pm.

In Scenario 1, we were primarily interested in the accuracy of our traffic classifier and AP performance prediction. We then focused on evaluating the relative *ranking accuracy* in Scenario 2. Let *B*_predict_(*φ*) and *B*_actual_(*φ*) be the predicted achievable throughput and the actual throughput of candidate AP *φ*, respectively. Let *φ*_select_ ∈ Ω denote the AP chosen according to a selection scheme given the set of *n*_*c*_ candidate APs, $\Omega =\{\varphi_{1},\varphi_{2},\cdots ,\varphi_{n_{c}}\}$. Then, the ranking accuracy is given by

$$\begin{array}{c} \begin{array}{c} \text{Ranking}\;\text{accuracy}=\frac{B_{\text{actual}}\left(\varphi_{\text{select}}\right)}{\max_{\varphi \in \Omega }B_{\text{actual}}\left(\varphi \right)} \end{array} \end{array}$$ (9)

where *φ*_select_ = arg max_*φ*∈Ω_ *B*_predict_(*φ*) in our scheme [12]. The ranking accuracy of UAAS was compared with those of two legacy schemes, SSF and Virgil [5], [15].

Referring to the Wifi-Reports scheme, we set the TTL of each historical information to three months because the network conditions typically change at timescales of months or more [12]. The required achievable throughput for AP selection, *δ*_*b*_, increased in the range [0, 20] Mbit/s. Other parameters were defined as follows: *δ*_max_ = 100 and $\delta_{\min}^{\left(t\right)}=40(\forall t\in A)$.

### 5.1 Prediction accuracy

To assess our traffic classifiers, we selected 100 pairs of the average and the normalized standard deviation measurements per application. Once an application had been determined to be tested, the measurements of the other 23 applications were used to train the classifier. For the NBK-based classifier, we made each type of kernel estimated density function, as presented in Section 4.2. For the BNN-based classifier, we utilized the BNN software package of Neal [30]. We used one hidden layer consisting of five nodes with no *bias* node.

**Fig 9:**
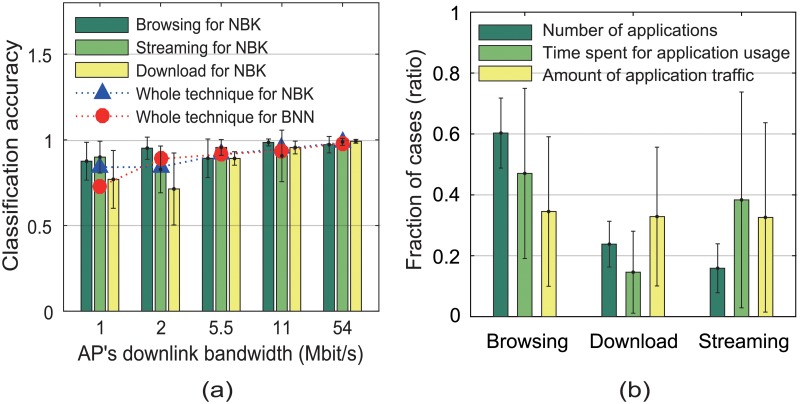
Classification accuracy and application usage patterns. (a) accuracy of our classifiers versus the AP’s downlink bandwidth and (b) the analysis of application usage for each type per user.

We define each type of classification accuracy as the probability that this type of application traffic is classified correctly. The whole technique accuracy is defined as the average of all types of classification accuracy values. Fig 9(a) shows that, for both classifiers, the whole technique accuracy improved with the increase in the AP’s downlink bandwidth. This is why our classifier operates when the user has been connected to the high-speed AP. At 54 Mbit/s, each type of application traffic was correctly classified with more than 97% accuracy.

We installed our monitoring application, WiNet, on the mobile devices of 70 users, and collected the information on every type of applications for two months. This information includes the package name, unique package ID, uplink/downlink traffic, and usage time of each application. Fig 9(b) shows that the most number of applications was classified as the browsing type and that the user spent the longest time using this type of applications. Nonetheless, we found that each type of applications generated a similar amount of uplink and downlink traffic.

**Fig 10:**
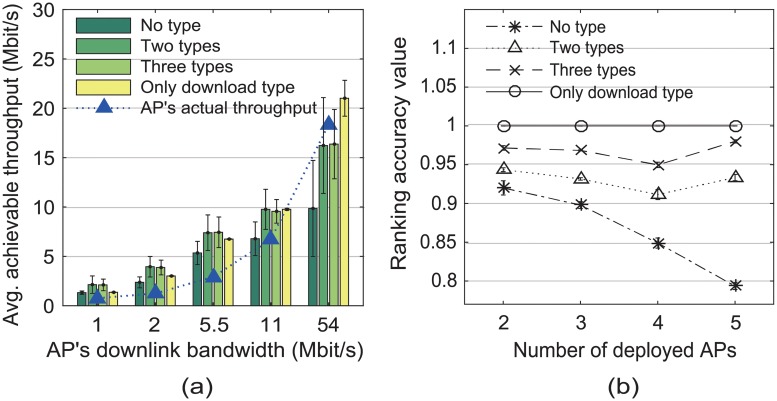
Achievable throughput and ranking accuracy in Scenario 1. (a) AP’s achievable throughput and (b) the ranking accuracy value corresponding to the application types considered to predict AP performance.

Fig 10 plots the predicted achievable throughput of the AP and the ranking accuracy value in Scenario 1. We estimated AP performance in the following four cases: 1) The application type is not considered, and AP’s achievable throughput is the average of all application traffic (no type). 2) Two application types are randomly selected to predict AP performance (two types). 3) All application types are considered (three types). 4) The download-type traffic is averaged as AP’s achievable throughput (only download type). We randomly collected the considered types of application traffic until the total number of measurements was more than *δ*_max_. To evaluate the ranking accuracy, we configured the number of deployed APs among the five APs used in Scenario 1 and tested the possible groups of consecutive downlink bandwidths. For example, if four APs were deployed, we tested two possible groups of consecutive downlink bandwidths as follows: {1, 2, 5.5, 11} Mbit/s and {2, 5.5, 11, 54} Mbit/s.

Fig 10(a) shows that, in the download only-type case, the variation in the predicted achievable throughput was small at every downlink bandwidth and the predicted throughput had a significant difference between different downlink bandwidths. This result means that the download-type traffic is the most reliable for predicting AP performance. Conversely, in the no-type case, the variation in the predicted throughput was large compared to its average value. Therefore, Fig 10(b) shows that the download only-type case achieved the best performance, whereas the no-type case had the worst performance with respect to the ranking accuracy.

### 5.2 Ranking accuracy

We evaluated the ranking accuracy of our scheme in Scenario 2. To this end, we simulated the application usage patterns of 70 users, which are summarized in Fig 9(b). Specifically, we measured the amount of traffic generated by an application package with a unique ID whenever the package was used and the amount of traffic generated by application packages included in each type in a location in one day, in the same method that was described in Section 3.2. We tested eight application packages per type and used their traffic collected in advance for each candidate AP. To consider the time of day, this application traffic was split into two measurements: 1) traffic collected during daytime and 2) traffic collected during nighttime. For every trial, we randomly selected a user from the 70 users, and the user generated all types of traffic according to his/her application usage pattern. Note that our scheme collects the performances of candidate APs in a location until it finds an AP whose achievable throughput has been predicted to more than the required throughput for AP selection, *δ*_*b*_. We ran 1000 trials for a given *δ*_*b*_.

**Fig 11:**
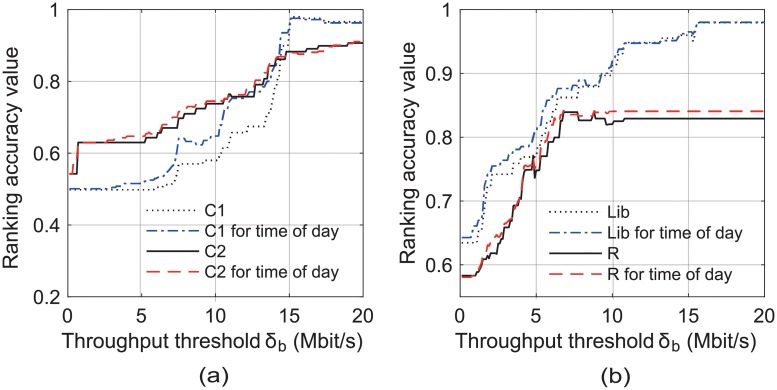
Ranking accuracy results of our scheme in Scenario 2. (a) at C1 and C2, (b) at Lib and R.

**Fig 12:**
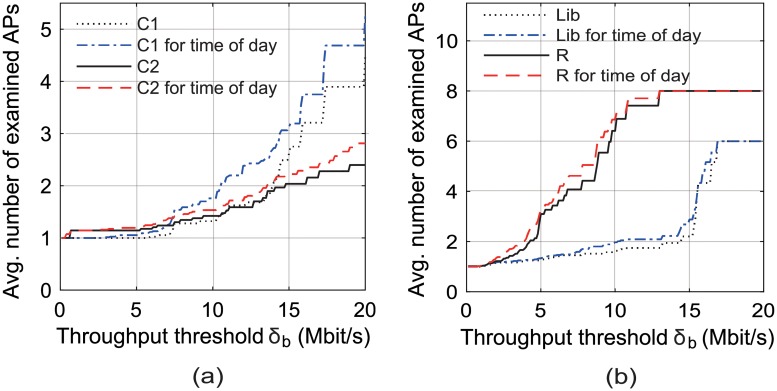
Number of examined APs vs. required throughput, *δ*_*b*_, in Scenario 2. (a) at C1 and C2, (b) at Lib and R.

Fig 11 illustrates the ranking accuracy of our scheme versus the required throughput threshold *δ*_*b*_ in the locations C1, C2, Lib, and R. We found that the ranking accuracy improved as *δ*_*b*_ increased in every location. This can be attributed to the fact that, as *δ*_*b*_ increases, there will be less number of APs whose achievable throughput is predicted to be more than *δ*_*b*_, and our proposed scheme selects an AP from among them considering their last access times and beacon SNRs. However, as shown in Fig 12, our scheme examines more candidate APs with the increase in *δ*_*b*_. Fig 11 also shows that the ranking accuracy increased as AP performance was predicted according to the application traffic split by the time of day.

**Fig 13:**
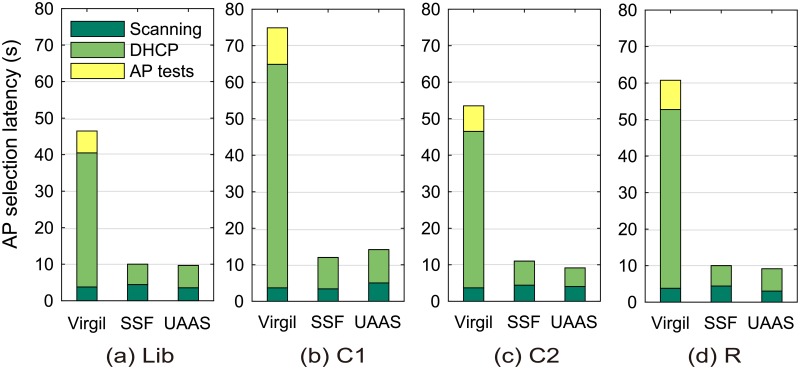
Average AP selection latencies of Virgil, SSF, and UAAS in Scenario 2. (a) at Lib, (b) at C1, (c) at C2, (d) at R.

Table 5 presents the ranking accuracy of two legacy schemes, Virgil and SSF, and the ranking accuracy of UAAS when the time of the day was considered and *δ*_*b*_ = 20 Mbit/s. Virgil was the optimal scheme with respect to the ranking accuracy, whereas SSF showed poor results in all the locations. This is because Virgil associates with every candidate AP and then measures its performance to find the best one. However, most commercial mobile devices cannot associate multiple APs simultaneously and, therefore, Virgil incurs a significant latency overhead. Fig 13 shows the AP selection latency of Virgil, SSF, and our UAAS scheme in Scenario 2. We found that Virgil’s latency was particularly high if a large number of candidate APs existed in the location. Conversely, our scheme does not introduce any additional latency as in the SSF scheme.

**Table 5:** Ranking accuracy values of Virgil, SSF, and UAAS.

	C1	C2	Lib	R
Virgil	0.80811	0.89319	0.78401	0.86345
SSF	0.48862	0.53133	0.63647	0.57716
UAAS	0.96304	0.91051	0.97996	0.84056

**Fig 14:**
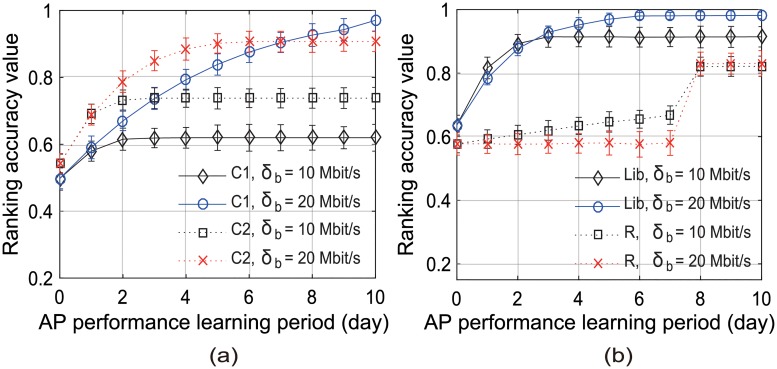
Ranking accuracy results of our UAAS scheme versus the period for AP performance learning in Scenario 2. (a) at C1 and C2, (b) at Lib and R.

Fig 14 shows the effect of the learning period on the ranking accuracy in Scenario 2. Our scheme needed (50 × *δ*_max_) traffic samples to examine a single candidate AP. We define the saturated learning period as the time between the initiation and the completion of the AP performance learning process. We found that the saturated learning period depended on the number of candidate APs and the required throughput for AP selection, *δ*_*b*_. For example, in C2, the saturated learning period was three days when *δ*_*b*_ = 10 Mbit/s, whereas it was six days when *δ*_*b*_ = 20 Mbit/s. We also found that, when no candidate AP was examined, the ranking accuracy values of our scheme were similar to those of the SSF scheme presented in Table 5. Conversely, when the learning period was long enough, our scheme outperformed the Virgil scheme.

### 5.3 AP switching policy

We used NS-3 to evaluate our switching policy. We deployed five APs, namely AP1-AP5, and AP2-AP5 were initially associated with 4, 3, 2, and 1 mobile devices, respectively. The number of mobile devices associated with AP1 was varied from 10 to 30. We assume that the achievable throughput of AP1-AP5 will be 3.4, 5.4, 7.4, 11.3, and 22.8 Mbit/s, respectively, when our scheme completes the learning process. Each mobile device generated 1000 download-type traffic samples per second and determined the switching when the measured achievable throughput was less than 2 Mbit/s; its maximum number of switchings was limited to four.

As described in Section 4.4, our scheme uses the probabilistic and delayed switching. For comparison, we define the *deterministic* and *immediate* switching. Once the performance of all APs have been collected, the deterministic scheme chooses the AP for which the highest achievable throughput is predicted; otherwise, it associates with the unexamined AP with the highest SNR. The immediate scheme promptly switches the association once it has selected a destination AP, whereas the delayed scheme postpones the switching by at most *D*_max_ = 8. We tested two situations: 1) the unsaturated learning situation, where each mobile device has collected the performance of only two random APs, and 2) the saturated learning situation, where the performance of all APs have been collected by the mobile device.

**Fig 15:**
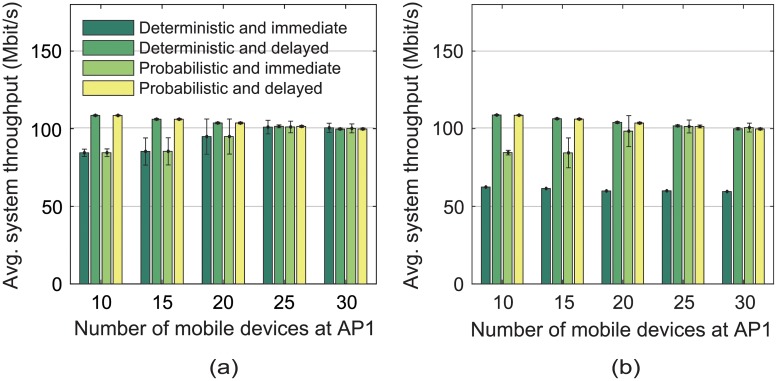
Average system throughput versus the number of mobile devices associated with AP1. When the number of APs whose performance was collected was (a) 2 (i.e., unsaturated learning) and (b) 5 (i.e., saturated learning).

**Fig 16:**
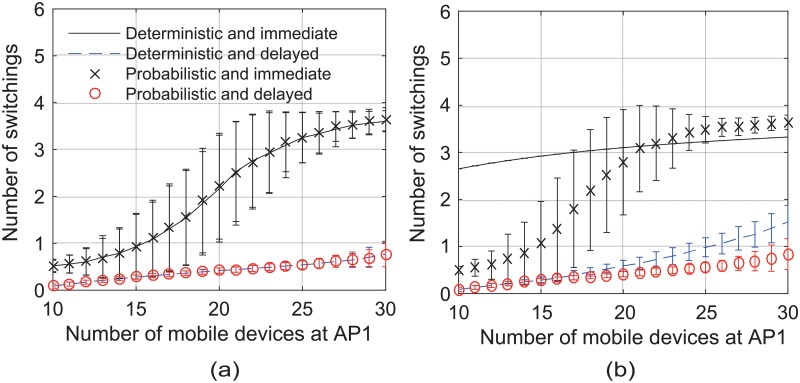
Average number of switchings per mobile device. (a) in the unsaturated learning situation and (b) in the saturated learning situation.

We define the system throughput as the total throughput of all mobile devices when their switchings no longer take place. Figs 15 and 16 show that the delayed scheme achieved higher system throughput and required fewer switchings, compared to the immediate scheme, in both the unsaturated and saturated learning situations. In the immediate scheme, when a serving AP became overloaded, its mobile devices began to find better candidate APs, and simultaneously switched their associations to a few chosen APs, i.e., destination APs; it soon degraded the performance of the destination APs, and the mobile devices had to switch their associations again. Conversely, the delayed scheme reduced the probability that a large number of mobile devices switch their associations to a few APs at the same time. Similarly, the probabilistic scheme required fewer switchings than the deterministic one without the loss of system throughput, as shown in Fig 16(b).

## 6 Conclusion

We found through the measurement studies that a user often detects tens of APs and there is a significant range in their achievable throughput in dense WLANs. However, the AP selection schemes based on received signal strength result in poor user experience in many situations. Other alternative schemes incur additional latency and signaling overhead in probing every single candidate AP in dense WLANs. To address this problem, UAAS monitors the amount of network traffic used by applications while the AP is being used. When the user re-encounters the AP, the historical application traffic is used to predict its achievable throughput. Our scheme improves the prediction accuracy by considering the characteristics of each type of application traffic. The results of our experiments using the measurements collected in actual dense WLAN environments and from actual mobile users showed that UAAS outperformed the previous schemes with respect to the achievable throughput and the association latency in the places highly accessible to the user. In addition, the simulation using NS-3 showed that our probabilistic and delayed switching policies achieved higher system throughput and fewer reassociations compared to the deterministic and immediate switching policies.
